# Primary prevention: a need for concerted action

**DOI:** 10.1002/1878-0261.12432

**Published:** 2019-01-18

**Authors:** Joachim Schüz, Carolina Espina, Christopher P. Wild

**Affiliations:** ^1^ International Agency for Research on Cancer (IARC) Lyon France

**Keywords:** cancer burden, cancer prevention, Europe, risk factors, tobacco control

## Abstract

The burden of cancer is increasing worldwide, and Europe is no exception in this regard. Cancer incidence rate for men in 2018, excluding nonmelanoma skin cancers, averaged over the 40 UN‐defined European countries has been estimated as 436/100 000. For women, the estimated incidence rate is 332.6/100 000. Although mortality rates are declining in most European countries, the total number of cancer deaths continues to rise due to an increase in the number of older people in the age range when the cancer typically occurs. The increase in incident cases and cancer deaths increases the pressure on healthcare infrastructure and related costs, thus presenting a challenge to health service sustainability in countries. In the general population, there remains a perception of an ever‐increasing cancer risk. Hence, treatment alone is not a solution to address the cancer burden. At the same time, recent estimates of preventable fractions of cancer suggest that about half of all cancer cases could be prevented through rigorous implementation of successful prevention measures, among other actions, by following the cancer prevention recommendations of the European Code against Cancer. Smoking alone explains almost half of all preventable cancers, and the scattered way of implementing tobacco control in Europe with still increasing numbers of lung cancers in women demonstrates the gap between prevention potential and effectively implemented prevention. Cancer prevention clearly needs more resources, stronger support from decision‐makers and society, and a solid network to better speak with one voice. The newly established ‘Cancer Prevention Europe’ (Forman *et al*., 2018) offers promising opportunities for the latter.

**Abbreviations**
CPE: cancer prevention Europe
DNA: deoxyribonucleic acid
EU: European Union
FCTC: framework convention on tobacco control
HPV: human papillomavirus
IARC: International Agency for Research on Cancer
NCD: noncommunicable disease
POP: persistent organic pollutants
UN: United Nations
UV: ultraviolet
WHO: World Health Organisation

## 1 Introduction

The burden of cancer is increasing worldwide. While the estimated total number of new cancer cases (excluding nonmelanoma skin cancers) was 14.1 million in 2012, it has been estimated to be 17.0 million in 2018 and is predicted to rise by 61.4% to 27.5 million in 2040 should this trend not be stopped or reversed (Ferlay *et al*., 2015; Bray *et al*., 2018; http://gco.iarc.fr/tomorrow/home). Respective global numbers of cancer deaths were 8.2 million in 2012 and increased to 9.5 million in 2018. Europe is no exception in this regard.

Mortality rates and their trends over time vary considerably by country and by cancer site. Where good healthcare facilities exist, cancer mortality rates are slowly declining, for instance by 1.3% overall in Europe over the past 6 years (Ferlay *et al*., 2013; Ferlay *et al*., 2018). From the mid‐1990s to 2010, this decline in cancer mortality has been more marked but less than for mortality from cardiovascular diseases, with just above 10% compared to 35% for the time period 2002–12 in the European Union (EU) countries (Malvezzi *et al*., 2018). Survival from cancer is characterised globally by a wide variation probably due to inequities in diagnosis and treatment (Coleman *et al*., 2008), with, however, limited representative data available outside Europe, North America and the more affluent Oceanian and Asian countries. Overall, improvement in survival is seen (Allemani *et al*., 2018), most likely attributable to a combination of greater cancer awareness, better early detection, better access to treatment and improvements in treatment itself. Nevertheless, 5‐year survival remains low for some common cancers even in wealthy countries, namely less than 15% for cancers of the lung or stomach, or even lower for oesophagus or pancreas (Dalton *et al*., 2008). Effects of the divergent trends in rates of cancer incidence and mortality are raising costs for early detection, treatment and after‐care given the increasing number of cancer survivors. Taking all this together, the spiralling increase in number of patients and costs of cancer care means that no country can afford to treat its way out of the cancer problem (Stewart *et al*., 2016).

In this review, we provide a more detailed look at the cancer burden in Europe, including its known causes as a first step in identifying goals of implementing cancer prevention. Following a discussion of barriers, we propose a way forward by more rigorous primary prevention strategies and joining forces across Europe. Ultimately, this has led to the foundation of ‘Cancer Prevention Europe’ (Forman *et al*., 2018), an international and multidisciplinary consortium of European research institutes, organisations and networks of excellence that has been created to develop world class prevention research in Europe to be translated into effective cancer prevention guidelines and policies at national and international level.

## 2 Cancer burden in Europe

For the 40 UN‐defined European countries, the totality of new cases has been estimated to reach 3.91 million in 2018 (excluding nonmelanoma skin cancer) with 1.93 million Europeans dying from cancer (Ferlay *et al*., 2018). As cancer occurs mainly in older ages, the major reason for this increase is the concurrent remarkable and pleasing increase in life expectancy. Life expectancy for a person born in 2017 is now 75 years in men and 81 years in women for Europe as a whole, somewhat higher in Northern, Western and Southern Europe with 79 years (men) and 83–84 years (women), and lower in Eastern Europe with, respectively, 68 and 78 years ( https://www.statista.com/statistics/274514/life-expectancy-in-europe/). Roughly, life expectancy in most of Europe has increased by about 5% over the past 15 years. Almost 11% of women and 7.5% of men were already 65 years or older in 2016 ( https://www.populationpyramid.net/europe/2016/); those are the ages where about half of all the cancers in Europe occur (Pilleron *et al*., 2018).

**Figure 1:**
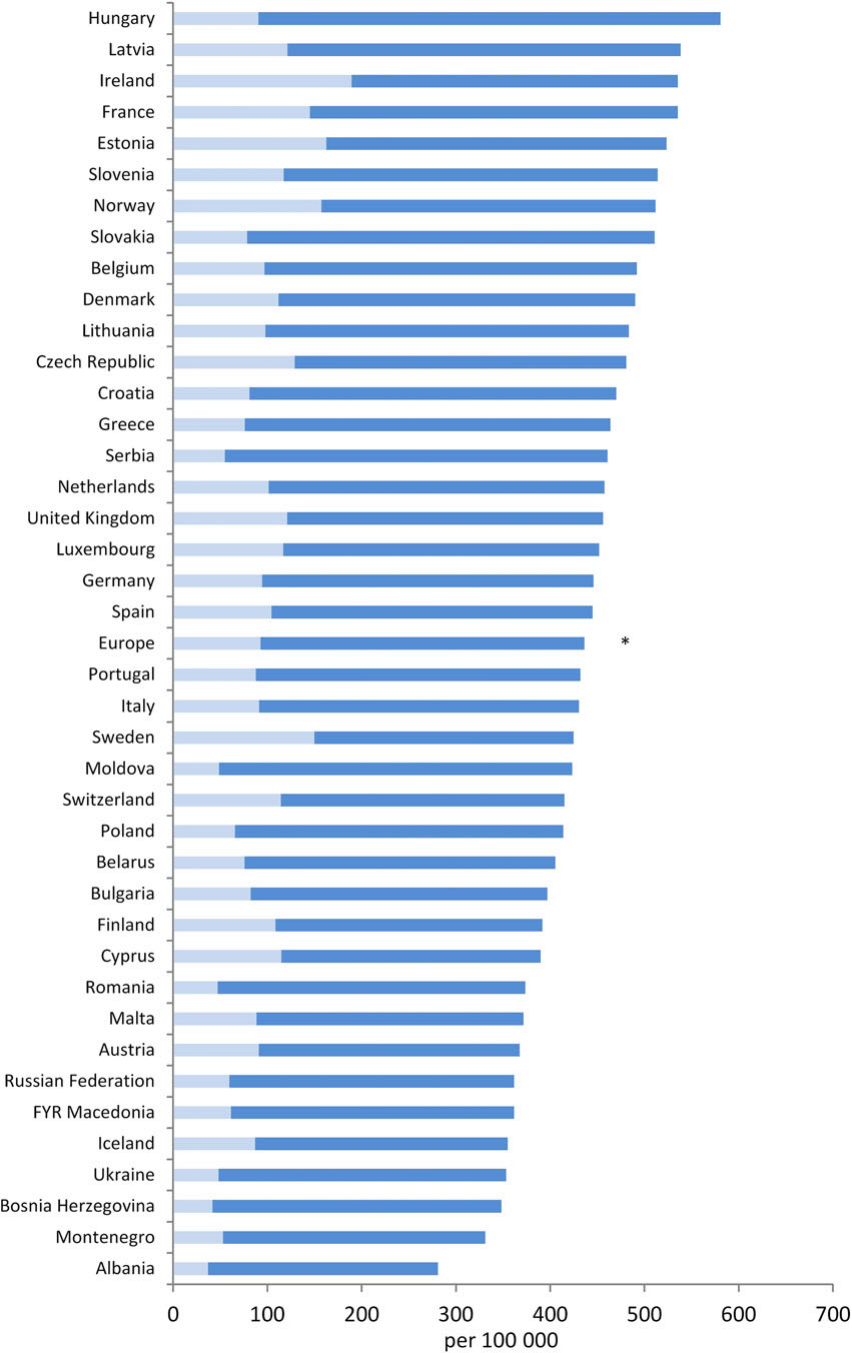
Estimated cancer incidence rates in European men (2018), age‐adjusted to European Standard Population (portion of prostate cancer shown in lighter colour).

**Figure 2:**
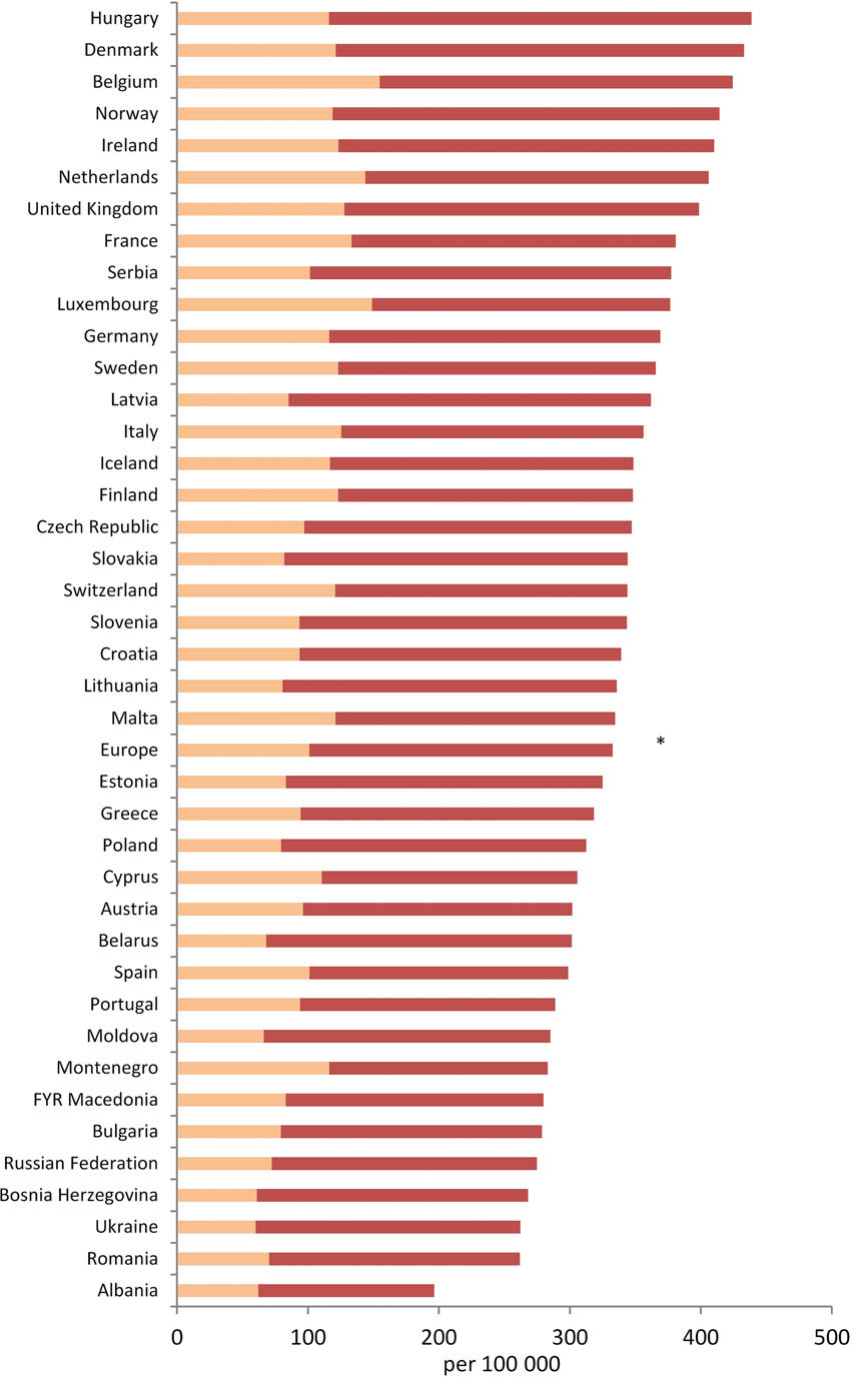
Estimated cancer incidence rates in European women (2018), age‐adjusted to European Standard Population (portion of breast cancer shown in lighter colour).

For men, cancer incidence – excluding nonmelanoma skin cancer – averaged over Europe in 2018 has been estimated to be 436/100 000, compared to 429.9 in 2012 (+1.4%; note some of the difference may have been introduced by differences in data sources at the two time points), with a factor of about 2 between the countries of highest (Hungary: 580.5) and lowest incidence (Albania: 280.6) (Ferlay *et al*., 2013, 2018); all country‐specific incidence rates are shown in Fig. 1 together with prostate cancer incidence rates, which, as the commonest cancer in men, is strongly influenced by screening (Ilic *et al*., 2013). For women, the European‐wide incidence rate in 2018 has been estimated to be 332.6/100 000, compared to 306.3 in 2012 (+8.6%), with – as for men – the highest rate being in Hungary (438.5) and the lowest in Albania (196.3) (Fig. 2; includes rates of breast cancer). For both sexes combined, the European‐wide incidence rate in 2018 has been estimated as 374.3/100 000 (+5.2% compared to 2012). Respective figures for cancer mortality are 165.8 (both sexes; −1.3% compared to 2012), 217.4 (men, −2.3%) and 128.1 (women, −0.5%). In summary, current European trends are therefore showing an increase in incidence rates, more pronounced in women, and a weak decline in mortality rates, slightly stronger in men. Combining this with the afore‐mentioned ageing European population, this results in a pronounced increase in the number of incident cancer cases (~ +13% from 2012 to 2018 for both sexes combined) and, albeit the declining mortality rates, a substantial increase in the absolute number of cancer deaths (~ +10%).

**Figure 3:**
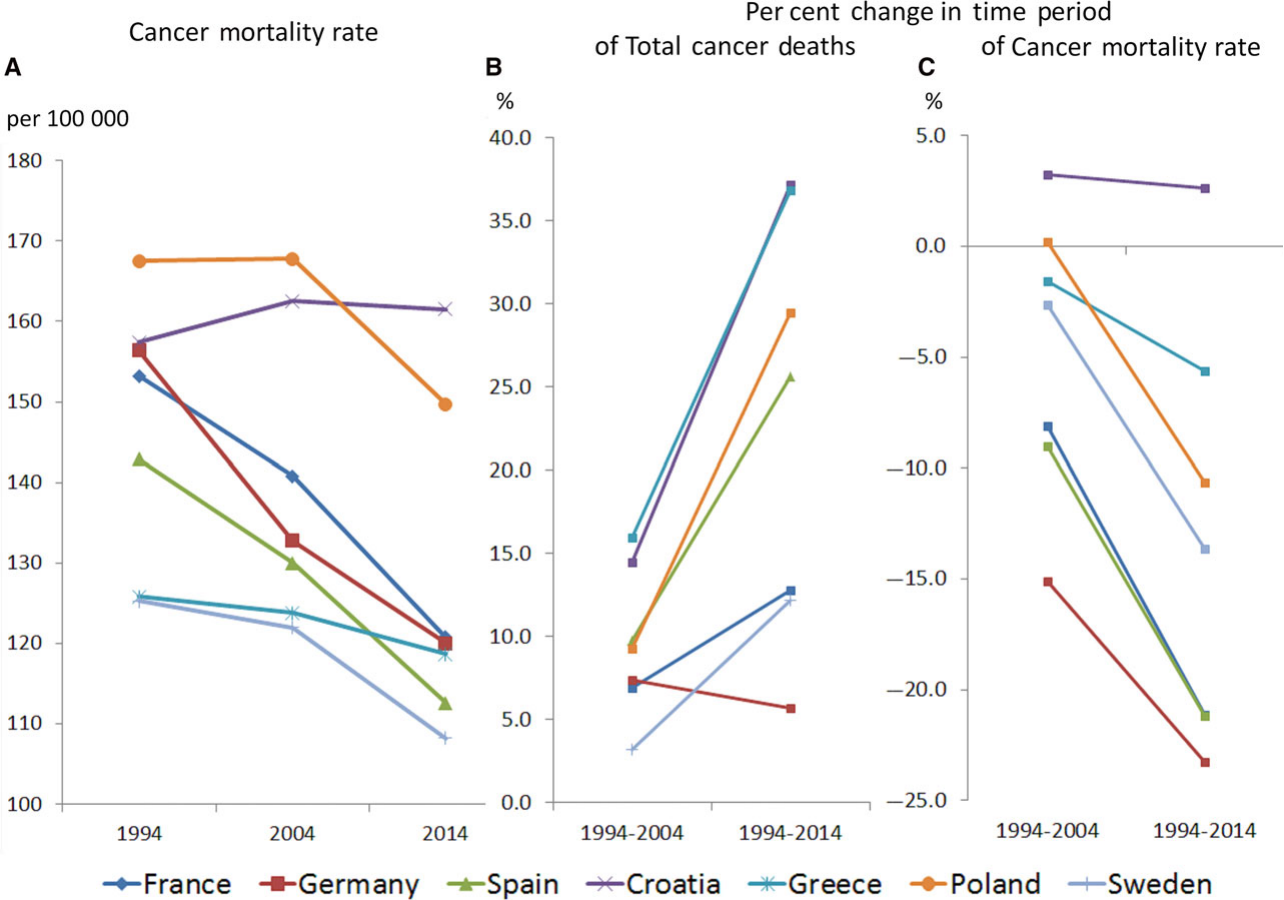
Time trends in cancer mortality between 1994 and 2014 in selected European countries; (A) Cancer mortality rate, both sexes combined, in 1994, 2004 and 2014, age‐adjusted to World Standard Population; (B) Per cent change in total number of cancer deaths for 1994 to 2004 and for 1994 to 2014; (C) Per cent change in cancer mortality rates for 1994 to 2004 and for 1994 to 2014, age‐adjusted to World Standard Population; (B and C) Both sexes combined.

Figure 3 illustrates this interplay between trends in risk, size and age of the underlying population, for seven countries from different parts of Europe for the 20‐year time period from 1994 to 2014 (WHO Mortality Database). Spain's >20% decrease in the cancer mortality rate corresponds to a > 25% increase in the number of cancer deaths. Hence, despite the success in reducing the risk of premature deaths from cancer, infrastructural demands for treatment and rehabilitation, and related costs to deal with the increasing numbers rise. In addition, dying from cancer will remain a growing concern from the population's perception, noting increasing cancer deaths among family, friends and other networks. From among the countries shown in Fig. 3, the increase in numbers reached 30% or higher in Poland, Greece and Croatia, but less than 10% in Germany. Mortality rates decreased in all countries except Croatia, the latter showing a modest increase.

Cancers of different sites and even of different histopathology or molecular signatures within the same site often differ in their aetiology. Therefore, for cancer prevention, even when aimed at reducing the total cancer burden, more detailed assessments by cancer site have to be done in order to optimise cancer‐specific interventions. In Europe in 2018, the top incident cancer sites in men were prostate (21.8%), lung (15.1%), colorectal (13.2%), bladder (7.5%) and lip, oral cavity and pharynx (4.3%), in contrast to lung (24.8%), colorectal (12.0%), prostate (10.0%), pancreas (6.0%) and stomach (5.7%) for cancer deaths (Ferlay *et al*., 2018). In women, respective figures were breast (28.2%), colorectum (12.3%), lung (8.5%), corpus uteri (6.6%) and skin melanoma (3.9%) for incidence, and breast (16.2%), lung (14.2%), colorectum (13.2%), pancreas (7.4%) and ovary (5.2%) for mortality. Among the common cancers, lung cancer is perhaps the one best understood in terms of risk factors, with the vast majority attributable to smoking and, to much lesser extent, several occupational exposures, air pollution and radon established as further causal risk factors (Cogliano *et al*., 2011); therefore – in theory – providing the largest prevention potential in numbers.

## 3 Modifiable risk factors (primary prevention)

**Figure 4:**
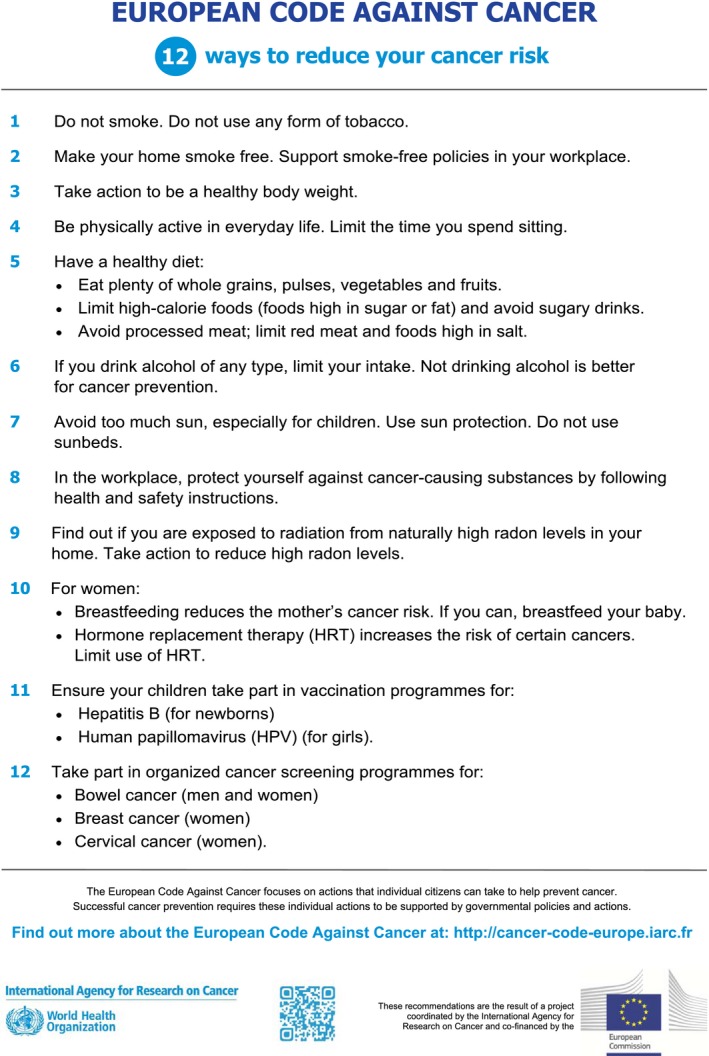
European Code against Cancer, 4th edition, 2014.

For Europe, it has been suggested that one third to half of cancer cases are preventable, as most of the established causes are exposures (including chemical, physical or biological agents) or unhealthy behaviours that are modifiable at individual or at population level or a combination of both (Schüz *et al*., 2015). Scientific evidence has been translated into a set of public health recommendations targeted to the individual summarising of what they can do themselves to reduce their risk of cancer, called the ‘European Code against Cancer’. This Code was first published in 1987 and updated in its 4th edition in 2014 (Schüz *et al*., 2015; Fig. 4). Notably, with for instance stopping smoking, maintaining a healthy body weight, being physically active, having a healthy diet and reducing alcohol intake, the individual has means to significantly reduce their cancer risk; nonetheless, all those actions should be encompassed in regulatory actions on for instance taxation and price policies on tobacco, alcohol or unhealthy foodstuffs, or urban policies to facilitate physical activity or ensuring sufficient shady places for solar radiation protection, for example in kindergartens or schools. Other smaller contributors to the cancer burden, but nevertheless established modifiable risk factors, are exposure to environmental pollutants or carcinogens in the work place, where action at a population level is required, such as for air pollution, safe work places or protection guidelines to eliminate or reduce exposures against harmful chemicals (Espina *et al*., 2015). Espina *et al*. (2013) reviewed successful policy frameworks for cancer prevention, related for example to asbestos, persistent organic pollutants (POPs), indoor radon, outdoor and indoor air pollution, second‐hand smoke, ultraviolet (UV) exposure including tanning devices and medical radiation; however, these frameworks need further strengthening.

**Figure 5:**
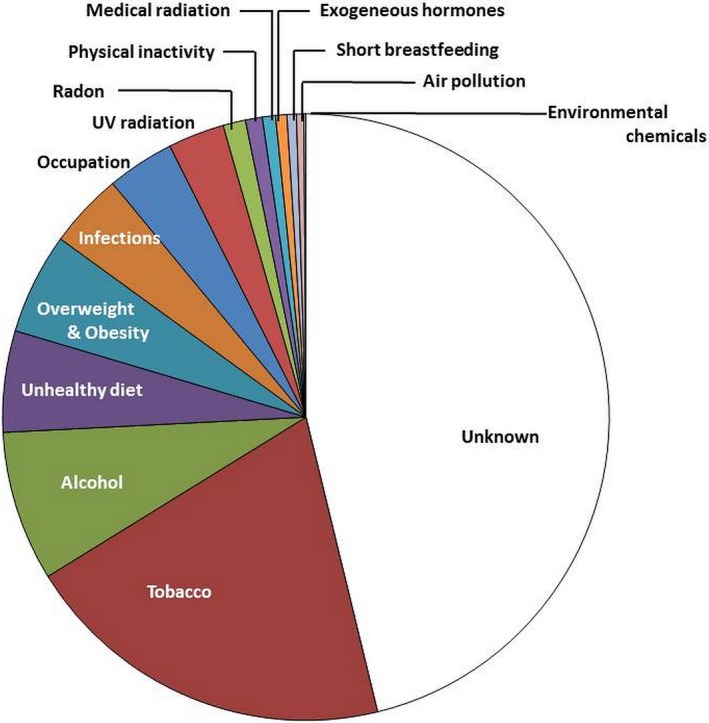
Attributable fractions of known causes of cancer, estimated for France ( http://gco.iarc.fr/resources/paf-france_en.php).

Recently, the contributions of different factors to the cancer burden have been quantified in France, suggesting that 41% of cancer cases are preventable ( http://gco.iarc.fr/resources/paf-france_en.php) as illustrated in Fig. 5; these results are supposedly broadly representative for many European countries. By far, largest contributor remained tobacco with 20% of the cancer burden and thereby causing almost half of all preventable cancers in France, followed by alcohol consumption with 8%. Other factors were unhealthy diet (5.4%), overweight and obesity (5.4%), infections (4%), occupational exposures (3.6%), UV (3%), ionising radiation (1.9%; radon and medical), lack of physical activity (0.9%), exogenous hormones (0.7%), no or shorter term breastfeeding [0.5%; breastfeeding reduces the mothers’ breast cancer risk (Scoccianti *et al*., 2015)], atmospheric pollution (0.4%) or environmental exposures to chemicals (0.1%). In parallel, another comprehensive calculation was performed for the UK (Brown *et al*., 2018), estimating similar impact by tobacco (15.1%), overweight/obesity (6.3%), unhealthy diet (4.8%), UV (3.8%), occupational exposures (3.8%), infections (3.6%), alcohol (3.3%), ionising radiation (1.9%), not breastfeeding (0.7%), exogenous hormones/oral contraceptives (0.6%) and lack of physical activity (0.5%). Noteworthy differences in comparison with France are the lower relative contribution from alcohol consumption (3.3% versus 8%) and the higher relative contribution by air pollution (1% versus 0.4%). It is interesting to compare these recent figures with a previous assessment for the UK (Parkin, 2011), applying exposure prevalence of around the year 2000. Tobacco was leading with 19.4%, followed by unhealthy diet (9.2%), overweight/obesity (5.5%), alcohol (4%), occupational exposures (3.7%), UV (3.5%), infections (3.1%), ionising radiation (1.8%), physical inactivity (1%), no breastfeeding (0.9%) and exogenous hormones (0.5%). Interestingly, those fractions changed less over time than one might have expected for one decade. Blot and Tarone (2015) however noted this before when reviewing the landmark publication on preventable cancers for the United States by Doll and Peto (1981), when in general estimates held true over a 35‐year time period (e.g. estimating 3% for occupational exposures). Lack of significant changes over time is likely due to a combination of slow implementation of primary prevention measures and the long time period elapsing between implementation and observable effects on cancer rates due to the long latency of most cancers between exposure and effect.

**Figure 6:**
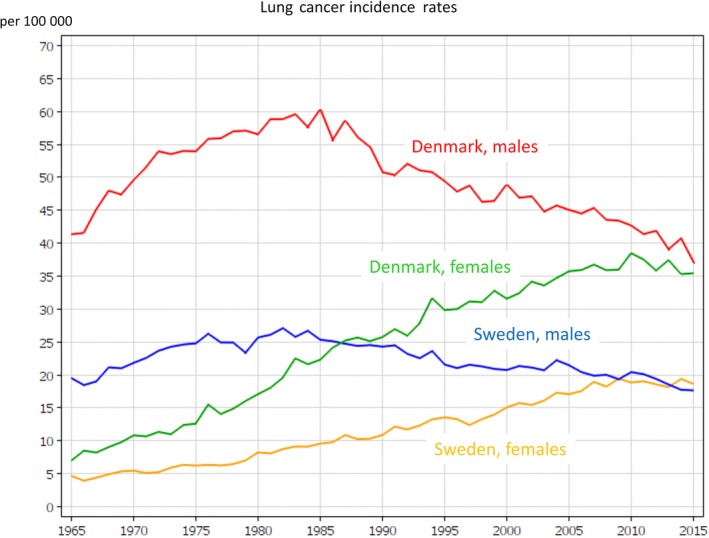
Time trends in incidence rates of lung cancer in Denmark and in Sweden, by sex, age‐adjusted to the World Standard Population, from 1965 to 2015 (produced with NORDCAN at http://www-dep.iarc.fr/;NORDCAN/english/frame.asp).

Smoking is approaching a century of being the cause of a lung cancer epidemic in Europe. It is by far the main contributor to the overall cancer burden, as illustrated by the lung cancer incidence time trends 1965–2015 from Denmark and Sweden, observed in two of the European countries with the longest history of accurate cancer registration (Fig. 6). It shows that among men, after all prevention efforts, lung cancer incidence in 2015 is about where it was fifty years earlier, whereas among women the steep increase over fifty years only recently seemed to reach a plateau. Sweden is the only country in Europe where the rates in women now exceed those in men, reflecting this unfortunate sex‐specific trend despite all the unequivocal knowledge on the harms of tobacco (Leon *et al*., 2015). Variation in lung cancer incidence rates of 2018 across European countries shows smoking is likely to remain the top‐ranked cancer cause for several years to come, with the incidence in men ranging from 111.6 (Hungary), 100.9 (Serbia) and 99.0 (Greece), to 65.2 (European average), to 25.6 (Sweden), 37.8 (Finland) and 40.0 per 100 000 per year (Switzerland) (Ferlay *et al*., 2018). The respective figures in women were 58.7 (Hungary), 53.8 (Denmark) and 48.1 (Iceland), to 26.4 (European average), to 8.2 (Belarus), 9.2 (Ukraine) and 10.5 (Albania). Overweight and obesity already show substantial contributions to the current European cancer burden and their increase to 30–70% overweight and 10–30% obesity proportions in adults ( http://www.euro.who.int/en/health-topics/noncommunicable-diseases/obesity/data-and-statistics) raise concern about an emerging epidemic (Arnold *et al*., 2015); therefore, effective approaches to primary prevention must be identified and enforced with immediate effect (Anderson *et al*., 2015; Peralta *et al*., 2018). Alcohol is an important target for more prevention efforts as awareness that alcohol causes cancer appears to be low in the population (Bates *et al*., 2018).

The contribution of infectious diseases to the total burden of disease in Europe, including cancer, is low due to past public health successes like the use of antibiotics, along with primary prevention strategies such as immunisation, access to clean water and safe food. Mortality from cervical cancer in many parts of Europe has been declining since the 1980s, mostly due to cancer screening and access to timely treatment; however, regional disparities still exist with high incidence rates in Central and Eastern countries of the EU that do not differ from those seen in parts of Sub‐Saharan Africa (Villain *et al*., 2015). The key element under primary cancer prevention of cervical cancer is human papillomavirus (HPV) vaccination, which is routinely provided in 33 countries in the WHO European Region (including all EU countries, except Bulgaria and Romania that recommend the vaccine for specific groups only), although with varied coverage rates ( https://vaccine-schedule.ecdc.europa.eu/ ; http://www.euro.who.int/en/health-topics/disease-prevention/vaccines-and-immunization/vaccine-preventable-diseases/human-papillomavirus-hpv2). A recent Cochrane evaluation has concluded that HPV vaccines protect against cervical lesions in young women (Arbyn *et al*., 2018). Likewise, the vast majority of Member States in the WHO European Region include hepatitis B in their immunisation programmes (some, e.g. Denmark and Finland adopt risk‐group‐targeted vaccination ( http://www.euro.who.int/en/health-topics/communicable-diseases/hepatitis/news/news/2017/04/hepatitis-b-vaccination-has-dramatically-reduced-infection-rates-among-children-in-europe,-but-more-is-needed-to-achieve-elimination).

Identification of successful primary prevention measures is a challenging and complex process of several steps. The first step is the scientific risk assessment (from hazard identification and dose–response assessment, to risk estimation and characterisation) which should be performed to appraise the potential impact of an exposure upon a defined population. The IARC Monograph program on the evaluation of carcinogenic risks to humans is seen as one of the most prominent programs on cancer hazard identification (Cogliano *et al*., 2011; Pearce *et al*., 2015). The program systematically reviews all the available scientific evidence concerning the carcinogenicity of an exposure (chemicals, complex mixtures, occupational exposures, physical and biological agents, and lifestyle factors), taking into account all lines of research, that is human studies (mainly observational epidemiological studies for cancer risk factors), animal bioassays and mechanistic studies. Subsequently, the risk characterisation process integrates all the information from the description of the hazard, dose–response assessments and exposure characteristics based on defined settings, to the estimation of the risk to individuals or populations in terms of the nature, extent and severity of the potential harms (including the fraction of the population likely to have developed a cancer because of a certain exposure). The second step is the risk management process, which makes use of the scientific risk assessment, in combination with socio‐economic and political inputs, to evaluate and select the most appropriate measures to manage the risks. During this risk management process, regulatory options are developed, prior to any decision‐making, to be considered by policymakers. After the chosen option is implemented, monitoring and evaluation of its effectiveness should be put in place. Finally, a cancer control plan should compile a set of these prevention and regulatory measures with the aim of reducing the cancer burden in the population.

While not in the scope of this review, we would like to emphasise the important role of secondary prevention in reducing cancer incidence and mortality. Organised screening programmes in Europe are recommended and exist for cervical, colorectal (men and women) and breast (women) screening (Armaroli *et al*., 2015), although unfortunately not everywhere and not fully harmonised (Basu *et al*., 2018).

## 4 Challenges of risk factor identification

With causes scientifically established for at present about half of all cancer cases, the other half remains unknown. It is widely accepted that cancer results from accumulation of genetic alterations that result in uncontrolled cell growth. Although the contribution of hereditary factors is recognised, the contribution of high penetrance genetic polymorphisms to the overall burden of cancer is limited in scale (Lichtenstein *et al*., 2000; Mucci *et al*., 2016). The vast majority of genetic alterations are somatic events arising from exposure to environmental factors or random mutational events associated with DNA replication (Klutstein *et al*., 2017; Nowak and Waclaw, 2017; Tomasetti and Vogelstein, 2015; Tomasetti *et al*., 2017; Wild *et al*., 2015). Distinct spatio‐temporal patterns in incidence rates of several cancers including those seen in migrant populations suggest that a significant portion of environmental or lifestyle causes of cancers may still be detected through additional research efforts. This emphasises the need for continued aetiological research in parallel to rigorously implementing interventions where available for preventable cancers.

There are several reasons why studies may have missed associations between environmental exposures and cancer risk: (a) observational studies, and especially those of case–control design required to estimate past exposures, use sometimes rather crude exposure measures used as proxies for complex exposure situations, having the potential to underestimate associations in particular at low doses; it is therefore conceivable that for instance chemicals known to cause cancer in occupational settings do so in the general population, even if not proven yet at dose levels to which the general population is exposed; (b) larger studies are needed to investigate potential interactions between factors with their co‐occurrence or sequential exposure causing cancer; (c) as epidemiological studies usually investigate risk of a defined ‘exposed’ population in comparison with a reference group considered ‘nonexposed’, the latter may not mean zero exposure for ubiquitous agents (such as natural radiation or air pollution), so studies would miss effects if levels occurring in the reference category are sufficient to lead to an increase in cancer risk; (d) there may simply exist further exposure–cancer combinations that have not yet been researched sufficiently well, for example of exposures during early life or multi‐causal pathways.

## 5 Barriers to overcome in primary prevention of cancer

The WHO noncommunicable diseases (NCDs) 2020 Action Plan is a good example illustrating how much knowledge on primary prevention of NCDs in general and cancer in particular exists, compared to how many open questions remain to be addressed before reaching the full extent of successful implementation (Diem *et al*., 2016). As mentioned above, it is important to acknowledge that primary prevention is not just changing individual behaviours in isolation, but requires broader changes in social, economic, political, environmental and cultural contexts. Undoubtedly, it needs capacity and resources, and public adoption of the measures, as well as multi‐sectoral action addressing the underlying, overlapping and interacting social determinants of NCDs (WHO, 2013). This becomes even clearer when reviewing the impact of the Framework Convention on Tobacco Control (FCTC; Chung‐Hall *et al*., 2018) to reduce the global burden of tobacco‐related disease. While measurable impact has been observed on tobacco consumption as a result of various measures, including price and taxation increases, smoking bans, tobacco marketing bans, health warnings, mass media campaigns to prevent smoking initiation and cessation interventions, acceleration of implementation is urgently needed; in particular, measures to counter industry inference, regulation of tobacco product contents, promotion of alternative lifestyles and protection of health and environment had lower implementation (Chung‐Hall *et al*., 2018). Similar conclusions were drawn in an evaluation of the WHO's MPOWER measures to reduce smoking‐related deaths, with 88 countries having adopted at least one of the six measures, for which increased cigarette taxes and comprehensive smoke‐free laws were estimated to have averted over 5 million smoking‐attributable deaths (Levy *et al*., 2018). On the other hand, as an example of outstanding challenges, tobacco surveillance in Italian minors over the past 20 years has shown only modest success in reduction in smoking prevalence, with declines in 11‐ to 13‐year‐olds but no decline in current and even an increase in daily smokers among 15‐ to 16‐year‐olds (Gorini *et al*., 2018), illustrating the need for stronger tobacco control measures in adolescents.

Political will for action, ideally with the pressure and support from society, is the main means for overcoming the barrier of current omissions in adoption of primary prevention measures. In times of information overload and confusing messages through different media, public health recommendations such as the ones included in the ‘European Code against Cancer’ are key tools in educating and empowering people to change individual behaviours but also to request support to put in place accompanying population level actions (Espina *et al*., 2018). In this context, to make informed decisions, information on what behaviours or agents are not established causes of cancer or unlikely to have major effects is equally important. Looking at the overall situation in Europe, there are indeed major efforts in various countries on primary and secondary prevention of cancer, but given the preventive potential of one third to half of all cancers, prevention today is still under‐developed and under‐resourced.

## 6 Role of cancer prevention Europe

To overcome barriers to prevention and with the aim of launching, evaluating and incrementally improving evidence‐based prevention strategies within Europe, an international consortium of European research institutes, organisations and networks of excellence has been created: Cancer Prevention Europe (CPE) (Forman *et al*., 2018). Covering a spectrum of research from behavioural and laboratory science to policy research, as well as dissemination of the best evidence, quality indicators and practices used, CPE will be broad in scope. A core component of the initiative will be endorsement of primary, secondary and tertiary prevention, as well as assessment of the cost‐effectiveness of different interventions, in relation to costs of treatment, care and productivity loss. Emphasis will also be placed on the research evaluation and advocacy dimensions of the prevention agenda. CPE will offer an integrated infrastructure capable of assuring high‐quality research. Each CPE partner institution will bring specific fields of expertise in cancer prevention research as well as in dissemination and informing policy and practice. The CPE agenda will include the following: ‘(a) research into optimising the implementation of known preventive strategies, (b) dissemination and research translation to inform policy and practice and (c) the identification of novel targets for prevention’ (Forman *et al*., 2018). Specific research areas are as follows: cancer registration; cancer aetiology (including recurrence); development and evaluation of preventive interventions (primary, secondary, tertiary); health economics and implementation research to enhance the effectiveness of intervention programmes. These will be supported by a range of platforms, networks and infrastructures and draw together a wide network of partners. Training and capacity building will be integral to the initiative. Successful coordination of cancer prevention requires long‐term vision, a dedicated research agenda and funding for such research, as well as a sustainable infrastructure and cooperation between countries and programmes. CPE offers the opportunity to fill gaps in the evidence base for prevention shaping the European cancer research agenda, to avoid common pitfalls in implementation and to share capacity for research training and quality improvement.

## 7 Conclusions

With the increases in life expectancy and population changes, if risk trends are not reversed, it is estimated that Europe faces an increase in annual numbers of incident cancers by almost 20% and in annual numbers of cancer deaths by almost 30% in the next 20 years (excluding nonmelanoma skin cancer). Even the wealthiest of European countries do not have the capacities to treat their way out of such an increasing cancer burden. It has been estimated that for Europe between one third and half of cancers would be preventable, if knowledge on successful prevention was transferred into rigorous action. While implementation science continues to evolve, for several key cancer risk factors, especially tobacco smoking still responsible for half of the preventable cancer burden, successful interventions are known but await stringent implementation. Reality however is that at present only a smaller part of preventive potential is used and further barriers need to be overcome, including obtaining support from health decision‐makers and greater advocacy from among affected populations. In Europe, stages of implementation of various measures are scattered, both for primary and secondary prevention, calling for joint efforts to overcome barriers. A voice for these urgently needed endeavours is the newly established ‘Cancer Prevention Europe’ (Forman *et al*., 2018; Wild *et al*., 2019), aligning with existing collaborative structures of leading cancer institutions, to develop strategies to translate basic, experimental, human and implementation science in cancer control strategies to effectively reduce the cancer burden.

## Author contributions

All authors contributed to the writing of this review article.
